# Medicaid Inmate Exclusion Policy and Infectious Diseases Care for Justice-Involved Populations

**DOI:** 10.3201/eid3013.230742

**Published:** 2024-04

**Authors:** Alysse G. Wurcel, Katharine London, Erika L. Crable, Nicholas Cocchi, Peter J. Koutoujian, Tyler N.A. Winkelman

**Affiliations:** Tufts University School of Medicine, Boston, Massachusetts, USA (A.G. Wurcel);; Massachusetts Sheriffs’ Association, Boston (A.G. Wurcel, N. Cocchi, P.J. Koutoujian);; University of Massachusetts Chan Medical School, Worcester, Massachusetts, USA (K. London);; University of California San Diego, La Jolla, California, USA (E.L. Crable);; Hampden County Sheriff’s Office, Springfield, Massachusetts, USA (N. Cocchi);; Middlesex County Sheriff’s Office, Woburn, Massachusetts, USA (P.J. Koutoujian);; Hennepin Healthcare Research Institute, Minneapolis, Minnesota, USA (T.N.A. Winkelman)

**Keywords:** Medicaid inmate exclusion, correctional health, infectious diseases, linkage to care, justice-involved

## Abstract

The Medicaid Inmate Exclusion Policy (MIEP) prohibits using federal funds for ambulatory care services and medications (including for infectious diseases) for incarcerated persons. More than one quarter of states, including California and Massachusetts, have asked the federal government for authority to waive the MIEP. To improve health outcomes and continuation of care, those states seek to cover transitional care services provided to persons in the period before release from incarceration. The Massachusetts Sheriffs’ Association, Massachusetts Department of Correction, Executive Office of Health and Human Services, and University of Massachusetts Chan Medical School have collaborated to improve infectious disease healthcare service provision before and after release from incarceration. They seek to provide stakeholders working at the intersection of criminal justice and healthcare with tools to advance Medicaid policy and improve treatment and prevention of infectious diseases for persons in jails and prisons by removing MIEP barriers through Section 1115 waivers.

Rates of illness and death from infections among justice-involved populations are high. Infections disparately affect persons incarcerated in correctional settings because of the syndemic relationship of infectious diseases, racism, and incarceration (*1*–*4*). In the early 1980s, high rates of HIV infection, hepatitis, and tuberculosis in correctional settings drew attention to missed opportunities to offer infectious disease testing and treatment (*5*,*6*). Correctional healthcare accreditation organizations, correctional administrators, public health officials, and clinicians have collectively advanced infectious disease care in correctional settings through investment into tuberculosis and HIV testing as well as HIV treatment and postrelease linkage programs (*7*,*8*). However, gaps persist, especially during transition from incarceration to community (*9*,*10*). Minoritized persons (including those who are Black, Hispanic, Indigenous, or sexually minoritized) are disproportionately incarcerated and particularly affected by lack of infectious disease treatment and prevention services in correctional settings and at re-entry into the community (*11*–*13*).

Resources allocated for infectious disease treatment and prevention in correctional settings are well documented as inadequate (*14*). Policy and financing reforms are needed to improve infectious disease prevention and treatment among justice-involved populations. The Medicaid Inmate Exclusion Policy (MIEP) prohibits federal Medicaid reimbursement for healthcare services delivered to any incarcerated person, except for hospital stays of >24 hours. Many states have applied to the federal government to waive MIEP through a Section 1115 Medicaid demonstration (hereafter referred to as the 1115 MIEP waiver) (*15*). We outline the history of MIEP, reflect on facilitators of and barriers to infectious disease care in correctional settings, and use the cross-disciplinary collaboration supporting application for an MIEP waiver in Massachusetts to highlight how infectious disease care paradigms could be positively affected by an 1115 MIEP waiver.

## Creation and Restructuring of the MIEP

In 1965, the Social Security Act created Medicaid as an insurance program to support access to healthcare for persons with limited income. The Social Security Act established the Inmate and the Institutions for Mental Disease exclusion policies to prohibit Medicaid reimbursement for services delivered in institutions, but it also allowed states to test new ways of delivering care through application for an 1115 MIEP waiver. In 1965, healthcare services available to persons living in the community were not routinely offered to incarcerated populations (*16*). In 1976, the United States Supreme Court ruled in *Estelle v Gamble* that correctional settings that failed to provide incarcerated persons with adequate medical care commensurate with the community-standard was a violation of the Eighth Amendment of the US Constitution. Although access to healthcare in correctional settings has vastly improved since then, wide variability remains (*17*,*18*).

Before the Affordable Care Act Medicaid expansion in 2014 (*19*), many persons released from incarceration did not meet their states’ Medicaid eligibility requirements, which often did not cover low-income adults without children. In states that expanded Medicaid under the Affordable Care Act, most persons became eligible for Medicaid at the time of release from incarceration; however, MIEP continued to prevent activation of Medicaid coverage during incarceration. In 1997, the Centers for Medicare & Medicaid Services (CMS) modified the scope of the MIEP by reinstating Medicaid coverage for incarcerated persons who are hospitalized >24 hours but continued to prohibit Medicaid coverage for outpatient services during incarceration (*20*).

## Barriers to Care Created by the MIEP

### Barriers to Infectious Disease Care during Incarceration

In 2011, an estimated one fifth of state department of corrections’ operational budgets was spent on healthcare (*21*). Even so, correctional budgets have been insufficient to meet the need, and the MIEP prevents Medicaid from filling this gap. For example, offering hepatitis C treatment to everyone who needs it has been challenging because of the cost (>$80,000 per treatment), a recommended treatment period of 8–12 weeks, and high rates of hepatitis C virus infection in jails and prisons (*22*,*23*). Other challenges for correctional healthcare budgets are paying for long-acting injectable medications that treat or prevent HIV infection and adopting substance use disorder treatments in jails and prisons (*24*,*25*). Many jails and prisons in the United States now offer medications for treatment of opioid use disorder and substance use disorder to prevent the risk for medical complications (e.g., withdrawal and death). However, medication continuity for opioid use disorder and many infectious disease conditions during and after incarceration remains poor (*26*).

### Barriers to Continuity of Care during Transitions from Correctional to Community Healthcare

Because Medicaid coverage is suspended or terminated during incarceration, it needs to be reactivated for persons to receive care when they return to the community. Most persons incarcerated in the United States spend short periods (<30 days) in jail (*27*) and often cycle multiple times from jail to community, further fragmenting needed care. People leaving jail and prison face barriers getting Medicaid reactivated, making appointments, and getting medications (*28*,*29*). Another barrier, with its own set of challenges, is data sharing between correctional and community healthcare systems (*30*). Virus eradication (hepatitis C virus) and virus suppression (HIV) are needed to end the hepatitis C and HIV infection epidemics, yet persons who leave jail and prisons with those infections often encounter administrative, geographic, and financial hurdles blocking access to treatment, further complicated by competing priorities of housing, food insecurity, and unemployment (*31*–*35*). Persons with untreated HIV infection (*36*), viral hepatitis (*37*), and substance use disorder (*38*) are particularly at risk for disjointed care when transitioning to the community.

## Cross-Disciplinary Collaboration in Massachusetts to Promote Healthcare Access and Waive MIEP

As of January 2024, at least one quarter of states, including Massachusetts, had applied for an 1115 MIEP waiver. In 2023, CMS granted 1115 MIEP waiver requests to California and Washington to cover transitional care services provided to persons in the 90 days before their release from incarceration (*39*), and CMS issued guidance to help states understand what provisions might be waived (*40*). In December 2021, Massachusetts submitted an 1115 MIEP waiver request with input from many collaborators, including but not limited to the Massachusetts Sheriffs’ Association and Department of Correction. As outlined in the waiver application, the major goals for Massachusetts are to improve prerelease and postrelease care management and connection to healthcare services, to improve healthcare outcomes, and to decrease outcome disparities (*41*,*42*). Incarcerated persons who meet Massachusetts Medicaid income eligibility criteria would be able to receive Medicaid covered services during a prerelease period. To ensure that all persons incarcerated within a facility have equal access to healthcare services, correctional budgets would need to support provision of Medicaid-covered services for persons who do not meet Medicaid eligibility requirements. Massachusetts originally requested coverage during a prerelease period of 30 days (*43*); the recent CMS guidance allowed a prerelease period of up to 90 days (*40*), and Massachusetts resubmitted its waiver request on October 16, 2023, proposing coverage 90 days before release for all incarcerated persons (*43*).

## Operationalization of an 1115 MIEP Waiver to Improve Infectious Disease Care

An 1115 MIEP waiver would provide several opportunities for improving infectious disease care. High-cost, evidence-based medications (e.g., for treatment for hepatitis C and preexposure prophylaxis for HIV) could be initiated before release and supported by robust linkage to care programs after release. Medications and treatment for substance use disorder could be augmented, reimbursed, and continued seamlessly in the community, enhancing opportunities for successful re-entry. Intensified support for care coordination and for linkage to care at the time of re-entry has also been proposed in the newest application—a strategy that has been shown to increase continuation of care and improve infectious disease outcomes (*44*–*46*). Care coordination staff embedded within the jail or prison would assist with completion of health insurance paperwork, scheduling of clinician appointments, and other tasks at re-entry. Data sharing between the correctional health system and the community health system would be improved. An 1115 MIEP waiver could change the experience of a person with an infectious disease or substance use disorder transitioning from correctional to community healthcare (Table).

**Table:** Case examples of potential impact of overturning MIEP on infectious disease care for eligible persons*

Case	Before waiver approval	After waiver approval
25-y-old man with HCV and opioid use disorder, incarcerated for 50 d, plans for release in the next month	• Short incarceration period and high medication cost are barriers to testing to confirm chronic HCV infection and to initiating HCV treatment. • Gap in insurance coverage impedes transfer of OUD treatment to pharmacy after release. • Interested in PrEP but no system to ensure follow-up by community clinician (community clinic requires active health insurance at time of appointment scheduling).	• HCV medications and PrEP initiated as soon as diagnoses are confirmed. • Minimum of 30-d supply of medications provided upon release. • With active insurance, appointment can be scheduled with community health center for day after release.
55-y-old woman with HIV and bipolar disorder, Incarcerated for 10 y and preparing for community re-entry in the next 2–3 months	• HIV diagnosed while in prison; does not have ties to a clinician in the community. • Bipolar disorder well managed in prison with medication; however, there are no systems to coordinate outpatient mental health care in the community. • She would like to connect with a community health center that can manage HIV and bipolar disorder. She does not know where she will be living, and she does not know which community health center will be accessible by public transportation.	• Linkage to care specialist connects with case worker to advocate for specific living situation near community health center. • Telehealth appointments scheduled with HIV clinician and mental health clinician before release to ensure warm handoff. • Phone number and appointment time for post-release appointment given to the patient. • 30-d supply of HIV medication and lithium delivered to living situation. • Phone number for care coordination contact at prison in case she has issues with medications or needs to transfer her care to a different community health center.
40-y-old trans woman receiving PrEP, incarcerated for 3 mo. Preparation for release began at intake.	• Has been receiving oral PrEP in the community, but PrEP not continued during incarceration. • Has not received STI testing or treatment in the community. The jail can do oral and urine STI testing; however, rectal testing is not available. • Interested in long-acting PrEP, but it was not on the jail formulary. • 1st hepatitis B vaccine given in jail but no plan for next vaccine	• Resources allocated from waiver funding to support protocolization of long-acting injectable PrEP delivery to persons within 30 d of release. • Infectious diseases nurse at the jails works with local clinicians and public health experts to coordinate testing for rectal gonorrhea and chlamydia. • Hepatitis B vaccine series scheduled at local pharmacy after release. • Records of vaccines and PrEP care transferred from jail to community health clinician.

*HCV, hepatitis C virus; MIEP, Medicaid Inmate Exclusion Policy; OUD, opioid use disorder; PrEP, preexposure prophylaxis; STI, sexually transmitted infection.

The 1115 MIEP waiver requested by Massachusetts would support a warm handoff, either through in-person or telehealth meetings, in which the clinician who will be treating the person in the community can meet with the jail or prison clinician. Medicaid enrollment during incarceration would enable providers to schedule appointments for persons soon after their expected release date; in some cases, the community provider might meet with the patient in person or via telehealth visit before release (*47*). The process of such handoffs is intended to reduce apprehension about stigmatizing experiences in the community and to improve engagement in postrelease care. The approach used by the Transitions Clinic Network, with 48 clinics across the country, serves as a model for hiring, training, and supporting a workforce dedicated to health at the time of re-entry (*48*,*49*).

As states implement MIEP-related policy changes, they should develop monitoring systems to help identify potential delays in healthcare access that may occur during incarceration or at the time of re-entry into the community. Moreover, states should establish accountability processes to ensure that correctional settings do not delay healthcare delivery until 90 days before release, when Medicaid could reimburse services rendered. For example, persons with liver disease from hepatitis C should be prioritized for treatment as soon as possible. Collaborative systems of care and open communication between clinicians, correctional administrators, and public health agencies should ensure that appropriate healthcare is delivered throughout incarceration and at re-entry into the community.

## Conclusions

Building on 1115 MIEP waiver–associated successes and lessons in California, Washington, and, eventually, Massachusetts, state Medicaid agencies can request to waive the federal MIEP to positively affect eligible justice-involved persons and the broader public. Repealing MIEP at the federal level would eliminate the need for states to apply for MIEP waivers. The growing number of 1115 MIEP waiver applications signals the strength of cross-sector partnerships among public health, policy, healthcare, and correctional leaders that can be leveraged for more robust legislative change to improve continuity of healthcare for incarcerated persons.
